# Racial/Ethnic inequality & contemporary disparities in mortgage lending

**DOI:** 10.1371/journal.pone.0308121

**Published:** 2025-01-14

**Authors:** Meghan M. O’Neil, Vincent J. Roscigno

**Affiliations:** 1 School of Criminal Justice, Michigan State University, East Lansing, MI, United States of America; 2 Department of Sociology, The Ohio State University, Columbus, Ohio, United States of America; University of Georgia, UNITED STATES OF AMERICA

## Abstract

Research over the past two decades has noted significant racial/ethnic wealth inequalities—inequalities with important implications for life chances and institutional access. Home ownership is as a foundational element of such inequality with broad consequences for exposure to crime, quality of public safety services, and access to healthcare, education, and employment. Building on earlier scholarship that has tended to focus on specific forms of mortgages, we draw in this article on over 1.4 million diverse mortgage applications from the largest 100 U.S. metropolitan areas to interrogate racial/ethnic disparities for (1) all home types (mobile homes, condominiums, multi/single-family units), (2) all lien holders (private/government backed), (3) all purposes (vacation/rental/owner-occupied), and (4) all buyer loan sequences (purchase, refinance, home-equity/improvement). Our analyses, which make use of multilevel modeling, reveal durable inequalities for African Americans and Hispanics across time and advantages for Non-Hispanic White and Asian-American applicants. Such disadvantages are likewise observed for those seeking housing in highly concentrated minority locales, although such effects seem to vary by applicant race/ethnicity. Specifically, mortgage originations, while generally less likely in high minority concentrated areas, appear to be more likely for Black/Hispanic borrowers in areas that have been becoming increasingly minority concentrated. Mortgage lending, we conclude, remains a deeply problematic dimension of racial/ethnic inequality with important consequences for persistent segregation, wealth disparities, and the intergenerational transmission of advantage/disadvantage.

## Introduction

As W.E.B. DuBois (1903) [1] accurately predicted, the color line of the 20^th^ century divided the nation and relegated African Americans and darker skinned minorities to a separate and unequal existence within institutional domains of housing, employment, health and well-being, education, and law. Importantly, the realities of formal and informal institutional exclusion and segregation also resulted in constraints in minority wealth accumulation [2–4]. In this article, we analyze an especially consequential dimension of wealth accumulation and intergenerational inequality, namely mortgages and access to home ownership.

Home ownership has long been a foundational source of family wealth and a significant gatekeeping mechanism when it comes to the best public services, neighborhoods, and schools [5–7]. This is especially important when one considers that disadvantaged neighborhoods with low rates of home ownership, especially those nestled in segregated urban areas, tend to experience high rates of criminal violence with broad implications for public safety and health and the intergenerational transmission of poverty [8–10]. Home ownership has also been linked to improvements in physical and mental health as well as access to capital to support healthcare costs later in life through reverse mortgages and home equity loans [11,12]. Some scholarship suggests that health gains may be smaller or elusive among minority homeowners [13,14] while other scholarship finds homeowners of color report higher levels of life satisfaction and fewer depressive symptoms relative to renters [15]. To what extent, given such implications, do we continue to find sizeable racial/ethnic inequalities when it comes to housing acquisition and access to mortgage lending specifically? This is the core question we address in this article.

As the great recession and foreclosure crisis made all too clear, minority loan access remains a pressing contemporary concern [16–18]. Predatory lending practices, especially those directed toward minority communities, were politically scrutinized and banks were subsequently pressured to restrict high-risk loans [19,20]. This likely had the effect of maintaining otherwise segregated housing patterns despite some diversity tied to Asian and Hispanic population growth [21]. Indeed, stable or shifting neighborhood compositions along with regulatory oversights may have altered the patterning of contemporary mortgage inequalities—a point that our analyses consider.

Building on prior work and drawing on a stratified random sample of nearly 1.4 million mortgage loan applications for the largest 100 metropolitan areas in the U.S., we analyze racial/ethnic inequalities in mortgage loan approval in 2004 (pre-recession period) and 2010 (post-recession period). We selected these not only given their connection to contemporary housing practices, but also due to the availability of robust data that can be supplemented with individual and place-based risk factors. We also consider the extent to which the neighborhood racial/ethnic composition matters either independently or in concert with the race/ethnicity of the prospective borrower. Our merging of The Home Mortgage Disclosure Act data, from which individual applicant data is derived, with American Community Survey locality data builds on prior work while also allowing for multi-level modeling and rigorous controls surrounding applicant background (i.e., income, loan-income ratio, loan type, gender, etc.).

Our analyses and discussion contribute to the social science literature in three important ways. Specifically, we: (1) offer analyses and a clear account of contemporary racial/ethnic inequality and its ongoing extent in mortgage lending; (2) interrogate whether such inequality has changed over time, possibly owing to shifts in regulation and government oversight; and (3) disentangle individual and neighborhood compositional effects and analyze their intersections. Importantly, our analyses also uniquely include distinct types of mortgage loans, including those (e.g., home equity, refinancing, etc.) that tend to be more exclusive and beneficial especially for applicants who are already economically advantaged. On this point, results indeed point to a shift between pre-recession and post-recession periods (once loan criteria became more restricted) in the types of loans applied for and granted, and with a greater share of mortgage loans being government backed and a greater share going to home equity and refinancing at lower interest rates. This suggests the possibility of minority disadvantage, to be sure, but also captures the reality that white and higher SES borrowers are able to take advantage of banking and lending practices when institutional screening processes become more restrictive. Without accounting for this, the extent of racial/ethnic inequality in lending will be significantly underestimated.

### Racial/Ethnic inequality and mortgage lending

The social science literature on racial/ethnic inequality over the last two decades has been quite clear that, despite the passage of Civil Rights legislation and fair housing gains, significant disadvantages persist that are rooted in everyday organizational processes e.g., [22,23], gatekeeper biases e.g., [24,25], and various forms of discrimination and race/ethnic hierarchy maintenance e.g., [26,27]. Home ownership and the mortgage lending that often precedes it are hardly exceptions. In fact, the processes underlying access to the mortgage market, including what some have described as “the fundamental whiteness of credit” [28], are rife with exclusionary and discriminatory potential [2].

Gatekeeping actors and white community backlash (i.e., white flight) have been at least partially culpable. Realtors, for their part, have historically engaged in race-based steering and in triggering white flight and panic selling. HUD’s Housing Market Practices Survey (HMPS), which conducted 3,264 audit tests in 40 metropolitan areas beginning in 1977, provided evidence of significant discriminatory steering of African Americans in sales and rental markets—a pattern that persisted between 1977 and 1989 according to two nationwide studies [29]. Although deemed unlawful with the passage of the 1968 Fair Housing Act/Civil Rights Act and decreasing somewhat since the 1980s, the steering of African Americans and Hispanics into minority concentrated neighborhoods persists [30–34].

The pre-application stage of home buying, long before one even takes out a mortgage, has also been historically problematic for minority communities given the federal government’s limited oversight/regulation of lending practices and refusal to insure or support loans, particularly in Black neighborhoods. Redlining, or the exclusion of entire neighborhoods from the infusion of mortgage capital, is a troubling and longstanding practice that undercuts the financial stability of Black and brown communities, as well as the larger metropolitan areas in which they are embedded. The health of a metropolitan area can derive from city-suburban integration, and to realize this integration, it is important to address the geography of opportunity [35]. Indeed, the federal government’s explicit refusal to insure mortgages in minority neighborhoods, beginning in the 1930s under the Home Owners’ Loan Corporation, solidified a race-based exclusionary process by circling black neighborhoods, refusing to insure them, and suggesting that the “risk” is too high for capital investment [36]. The resulting undercapitalization of minority neighborhoods is well-known and, despite civil rights legislation and regulatory changes aimed at dispersing capital in a more equitable manner, minority communities continue to fare poorly.

Lending itself—the focal point of our analyses—has evolved in a somewhat parallel vein. In prior eras, race/ethnicity was explicitly considered during the application process. Although technically no longer the case, contemporary algorithmic may be playing a role, more or less, by underwriting things like neighborhood crime rates, single-parent households, and foreclosure rates, all of which undercut mortgage origination and approval for prospective minority homebuyers e.g., [17,37,38]. Indeed, according to some recent work, such algorithmic underwriting may very include variables that, in essence, act as racial proxies, thus contributing to racial and place-based disparities in subprime lending [39]. We do, however, recognize that algorithmic processing of applications may also reduce racial disparities through standardization even though it cannot eliminate discrimination in loan pricing [40]. Indeed, the introduction of algorithmic filtering, which is less commonly utilized in neighborhoods with high rates of minority borrowers, likely excludes race and ethnicity on applications, at least relative to traditional underwriting. More research, however, is warranted if one is to definitively assess such inequality producing verse reducing possibilities of algorithmic processing [41].

Regardless of algorithmic lending practices in particular, inequities tied to steering and/or financing have historically tended to stymie minority home purchases and relegate prospective Black and Hispanic homebuyers to a smaller pool of homes in more heavily concentrated, under-resourced, minority neighborhoods. Segregated neighborhoods with lower-quality housing collide to increase long-term risk and reduce attractiveness to mortgage lenders. Indeed, and on this point, the mean and median value of minority-owned homes is lower than White-owned homes [42]—a pattern that lends itself to depreciation or loss of home wealth owing to the quality of the house and neighborhood and/or the denial of home improvement loans [43,44]. In considering such patterns and their contemporary pertinence, we expect most generally that:

Hypothesis 1: Minority applicants for home mortgages in the contemporary era will be more likely to be turned down than White applicants, even when controlling for arguably important individual applicant attributes.

Importantly, we already know from prior work that if and when African Americans receive mortgage loans such loans are more likely to be predatory and subprime [17,36,45–50] and are more likely to carry higher interest rates and risk for default [51,52]. The practice of distributing the higher cost mortgage products to African American and Hispanic borrowers was exacerbated post foreclosure crisis relative to Non-Hispanic White borrowers [46]. Among Hispanic borrowers, disparities have been observed with black Latinos experiencing the most loan rejections and the highest cost mortgage originations relative to white and Asian Latinos [50]. While we recognize that the topic of subprime lending and contracting of the subprime market is an important one [45–50], our research aim is not to capture mortgage rates and terms, but rather, to contribute to literatures on mortgage outcomes across race/ethnic groups and for more types of homes, e.g., multifamily, single family, manufactured and more types of loans, e.g., USDA Farm Service Agency loan, The Department of Veteran’s Affairs loan. Doing so offers a more inclusive and expanded understanding of equity or inequity in the US mortgage market.

Partly driven by the fact that Black homebuyers are less likely than Whites to have networks that can help with a down payment (10% for African Americans versus approximately 46% for Whites) and have less equity to fall back on [53], minority homeowners are also more vulnerable to an array of financial shocks [54–56] and, thus, a return to renting compared to Whites [56–58]. One such shock surrounds the economy, economic recession, and resulting income precarity. Hispanic borrowers have also been subject to a higher risk of housing loss and foreclosure when purchasing homes in segregated neighborhoods and in particular states (e.g., California, Nevada, Arizona, and Florida) where markets experienced extreme booms in housing values prior to the crisis [59].

### Recession, precarity and implications for racial/ethnic inequality

The pre-recession mortgage market of the 1990s and early 2000s, including the expansion of predatory lending, was partially driven by the development of derivative investments (i.e., collateralized debt obligations and mortgage-backed securities). Mortgages, which historically flowed directly from banks to borrowers, became indirect financial vehicles, originating with brokers before being sold off to investment banks where they were restructured into mortgage-backed securities. This had ripple effects that reverberated throughout the mainstream economy during the Great Recession of 2007–2009, and regulators and legislators began to scrutinize such practices more closely, especially securitization—i.e., the pooling of many mortgages together, including high-risk mortgages to underwrite bonds [60].

It was this aggressive packaging and selling of high-risk mortgages via securitization that fueled the exploitation of Black and brown households—households that, on average, have lower financial literacy, lower wealth reserves, smaller amounts of intergenerational financial support, lower credit scores, lower incomes, and higher propensity to experience significant shocks (e.g., incarceration, unemployment, divorce and bankruptcy) [53,61–64] The risk that would have historically been held onsite by banks and measured against the bank’s deposits—which discouraged making bad loans—seemingly disappeared as mortgages were bundled into securities and fee-based rating systems that artificially rated otherwise risky mortgage-backed securities as low risk.

Although the cross-sectional character of our data precludes consideration of specific political mechanisms (e.g., shifts in regulation and oversight) that may have altered racial/ethnic inequalities across time, there is good substantive and historical reason to expect some aggregate shifts in both inequality and the share of certain types of mortgages between the pre- and post-recession periods. For instance, increased government scrutiny, such as the Dodd-Frank Wall Street Reform and Consumer Protection Act that followed the mortgage boom and bust of the Great Recession was arguably aimed at reining in risky and exploitative lending and level the playing field for prospective minority homebuyers and those seeking mortgage capital in minority neighborhoods [60]. There was arguably also a tightening of standards, greater scrutiny of financial wherewithal, employment records, and credit scores, and a sharper institutional focus on predicting (and avoiding) risk. While the year 2010 presented an ongoing financial crisis, rather than a post-crisis era (e.g., high unemployment rates, alongside quantitative easing, and weak fiscal policy) it marked a shifting mortgage lending environment.

Our data—discussed in more detail momentarily—speak to the points above, at least indirectly. Table 1, for instance, shows a significant decline in overall loan applications across pre- and post-recession periods and a significant and proportional shift in whether loan originations were made in the private (and, to some extent, subprime) sector versus government-guaranteed (i.e., backed by the federal government, such as FHA or Veteran’s loans). One plausible result, given closer scrutiny of applications, may have been reductions in racial/ethnic inequalities in loan origination over time. Specifically:

Hypothesis 2: Owing to greater scrutiny, reductions in predatory lending and greater reliance on supposedly neutral criteria, racial/ethnic inequalities in mortgage loan originations might have declined significantly in the post-recession period.

**Table 1:** Total distribution of all mortgage loan applications and shifts between pre- and post-recession periods for our randomly generated sample.

2004		2010	
Private	Government-Backed	Private	Government-Backed
921,309 (95.565%)	42,760 (4.435%)	303,831 (80.065%)	75,651 (19.935%)
**Total: 964,079**		**Total: 379,482**	

We say supposedly given what we know about unequal employment histories stemming at least partially from ongoing racial discrimination hiring and firing e.g., [24,25,65] and the unequal long-term accumulation of wealth and credit [3,66]. Such criteria are hardly race neutral even in the algorithmic computational sense [67,68]. By loan origination, we are referring to applications for housing loans that make it through the entire process and that are fully funded. There are multiple processes wherein such applications might be derailed and wherein bias and inequality can come into play. Focusing on differential rates and inequalities in origination, however, is especially useful for our purposes and captures the overall extent of inequality in the mortgage seeking process. Prior research is informative yet mixed on this possibility. One study looking at disparities across the largest 100 metropolitan areas, [69] for instance, described mostly persistent minority disadvantage over time. The principal focus in this earlier analysis centered largely on neighborhood compositional changes and their possible impact. Our current focus is more directly on group inequalities in loan approval and intersections with neighborhood composition. We also interrogate more systematically the possibility of shifts in mortgage application types and approval across time.

**Fig 1:**
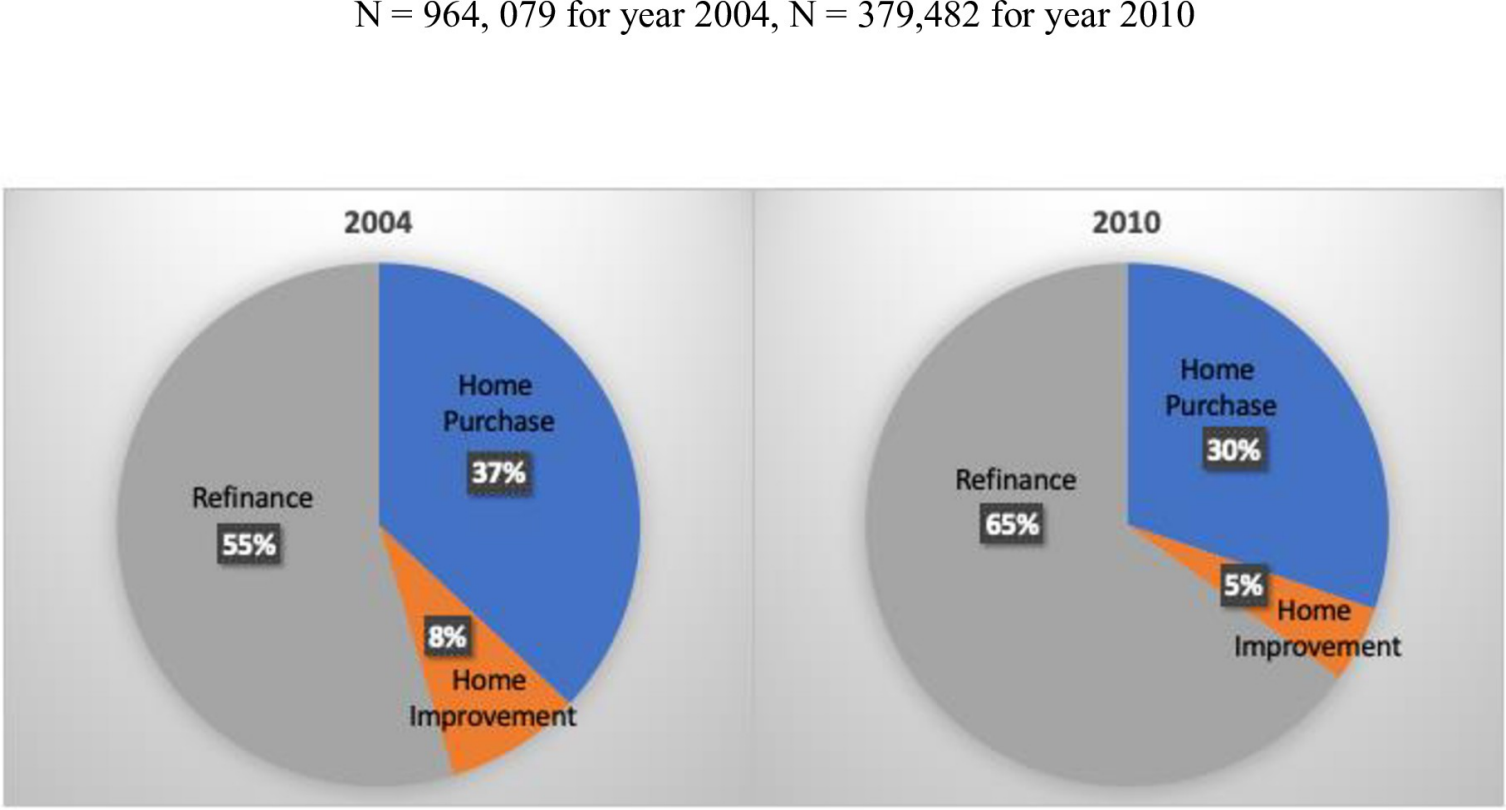
Shifts in mortgage loan application and approval by type, pre- and post-recession.

In a more recent analysis, some scholars similarly look at pre- and post-recession periods [50]. They restrict their analyses, however, to owner-occupied single-family home purchases specifically. In this regard, they find (relatively consistent with Hypothesis 2) that inequalities for Black and Hispanic mortgage seekers seem to have dissipated somewhat following the Great Recession. This is perhaps tied to heightened levels of lender scrutiny. Importantly, however, the exclusion of other forms of mortgage loans (e.g., government-backed, refinancing, second mortgages, rental properties, mobile homes/trailers) in their analyses likely leads to important underestimates in the overall extent of racial/ethnic inequality. Indeed, when comparing pre- and post-recession mortgage seeking and approval, one finds sizeable proportional shifts. These shifts include movement of applications and approvals away from owner-occupied, single-family home mortgages and purchases toward both higher scrutiny government-backed loans (see Table 1, earlier) and a greater representation of refinancing applications and approvals (Fig 1). Since these arguably put Whites and those of higher SES background at a heightened advantage, it is essential to analytically consider such mortgage types when drawing conclusions about racial/ethnic disadvantage and advantage.

Along with shifts toward government-backed loans and refinancing, the expectation that racial/ethnic inequalities in overall mortgage lending may have been exacerbated rather than reduced over time is further derived from recognition that: (1) loan criteria in the post-recession period became increasingly restrictive, with potentially disproportionate consequences surrounding the exclusion of minority loan seekers; and (2) there has been a growth of algorithmic underwriting of housing appraisals and financial approvals that could, in fact, reify rather than ameliorate racial/ethnic inequality [39,67,70]. In this regard, some bodies of recent work highlight ways in which inequality is intensified or at least maintained through such seemingly neutral algorithmic calculations e.g., [67,68,71,72] and everyday organizational processes [22] in a way that can disadvantage minority communities. We cannot directly test for such processes in our analyses given the cross-sectional nature of the data; nevertheless, suspect that the growth in algorithmic underwriting practices widens racially disparate outcomes for mortgage seekers. Recent work on the restrictive character of loan approval and likely bias even in face of neutral-appearing processes nevertheless leads to an alternative expectation to Hypothesis 2 about change over time. Specifically:

Hypothesis 3: Tighter scrutiny, more sophisticated algorithmic considerations, and the drying up of the subprime market during the post-recession period likely reduced the allocation of mortgage capital to minority applicants and communities and, thus, increased or at least maintained observed levels of racial/ethnic inequality in mortgage origination over time.

### Neighborhood composition & potential intersections with borrower race/ethnicity

Our analyses also importantly consider racial-ethnic compositions and compositional changes in the localities in which loans are requested and whether they have baseline or conditional effects on the likelihood of loan origination. This is important, according to prior literature. Prior work on White flight, for instance, details how racial/ethnic composition shapes perceptions of neighborhood desirability and buying/selling decisions [73–78]. Loan originations, we believe, may similarly be impacted for several reasons.

Minority presence and population growth within a given locality, prior evidence has shown, can trigger rapid ethnic turnover—turnover that prompts Whites to leave and discourages other Whites from moving in. The result over time can be lower desirability, racial homogeneity [77,79], and the creation of an unstable residential environment [29,80–84]. In these regards, other scholarship [85] has found that minority population concentration is associated with diminished public resources, including public safety and school quality—a pattern that reifies the socioeconomic status of a given neighborhood. Lenders and appraisers most likely downgrade housing in such contexts, reducing the likelihood of loan origination and approval, albeit perhaps less so if the race of a mortgage applicant matches that of the neighborhood [79].

Consistent with these points is the finding in most prior studies that large and/or increasing Black and Hispanic populations decrease home values [86–88]. Concentrated Black neighborhoods, research shows, have lower credit scores, on average, higher vacancy rates, and lower proportions of owner-occupied housing [89–92]. Higher rates of housing loss (return to renting) and serious mortgage delinquency and foreclosure are also more commonplace [93–95]. This both undermines the appreciation and resale potential of minority-owned homes [43,54,75] and, no less important, shapes lenders’ risk calculations and decision-making [96–98].

Potential associations between neighborhood racial composition and mortgages, while probably most relevant to prospective African American homebuyers, may also be consequential to Hispanic and Asian applicants given the notable growth of these populations over the last few decades [99]. Among Asian borrowers, subprime mortgage products were more common in nonwhite neighborhoods, however to a much lesser degree than African American, Hispanic, and even Non-Hispanic White borrowers [47]. Recent work in this regard suggests some parallels especially with regard to local Hispanic population growth [100]. Whites’ distaste for residing among minority neighbors, including Hispanics, can trigger discrimination among actors in the mortgage market and among realtors and lenders [34,44,101]. Asians, for their part, are significantly less segregated from Whites [99,100,102,103], are less likely to become a significant share of a given neighborhood given overall lower representation in the U.S. population [104] and, on average, resemble or exceed Whites on several key socioeconomic criteria, such as credit scores and income [52]. Following these points, we expect the following:

Hypothesis 4: The likelihood of mortgage loan origination will be diminished for applicants seeking housing in high and/or growing minority concentration (particularly African American and Hispanic) neighborhoods. We caveat this expectation, however, with the possibility that such effects surrounding minority composition may be conditional on the “match” with applicant race/ethnicity.

## Materials and methods

A unique and comprehensive dataset at both applicant and neighborhood levels was compiled for our analyses. We sought to overcome the limitations of many prior scholarly contributions that focused solely on one or a small number of marginalized urban communities by examining a rich representative sample of America’s largest 100 metropolitan areas [105]. First, Home Mortgage Disclosure Act “HMDA” files for all federally reported mortgage applications made during the years 2004 (pre-recession) and 2010 (post-recession) were downloaded from the Consumer Financial Protection Bureau, totaling 61.1 million applications [106].

A stratified random sample of 10 percent of cases in 2004 (n = 964,079) and 2010 (n = 379,482) across the largest 100 metropolitan areas in the U.S. was selected for inclusion. We utilize a sample of mortgage applications, rather than the entire population, in an effort to maintain computing power given the breadth of the data, including for instance, over 6.7 million mortgage applications for just the year of 2010. We began with the population of all federally reported mortgage applications nationwide during the years 2004 and 2010. Separated by year, we then drew mortgage applications from the top 100 metropolitan areas by population and generated a random ten percent sample using Stata. We follow past precedent and previous scholarship in our selection of a 10% stratified random sample with national HMDA data, see [38,69,107]. Importantly, we also engaged in sensitivity analyses in this regard by creating three 10% stratified random samples and interrogating both descriptive statistics and regressions for each to ensure comparability. Each 10% random sample produced overwhelmingly similar results, thereby increasing confidence in our overall sampling strategy.

Individual applicant data, which include the outcome of the loan origination process, race/ethnicity, gender, and other individual attributes, were then matched with 1990, 2000, and 2010 publicly available neighborhood level (census tract) data from the US Census Bureau and the American Community Survey (ACS) [108,109]. Our operationalization of key indicators is discussed below in more detail. Our matching of individual applicant and neighborhood data allows for analyses of neighborhood change over time and potential effects on loan origination. Data for the year 2000 is interpolated to 2004 to match mortgage applications, whereas the 1990 and 2010 Census data allow for the calculation of racial/ethnic compositional change over the periods of interest. The richness of our data is magnified with the inclusion of supplemental files providing area level credit scores as well as the creation of individual level risk ratings like price-to-income ratios. The years of 2004 and 2010 were selected due to the importance of historical economic shocks which produce practice and policy shifts for ongoing mortgage practice, as well as because we could explore robust data with individual and place-based risk factors incorporated that are representative of the largest 100 metros nationally situated in standardized tract boundaries. We utilized the Longitudinal Tract Data Base (LTDB) to harmonize census tracts from each year to 2010 census tract boundaries [110]. Lastly, we use multiple imputation techniques to handle missing data on our independent variables.

### Mortgage applications and loan origination

Our central outcome of interest is mortgage origination. Mortgage applications that qualify as originated are those that were approved and made it through the process of being fully funded. Given that racial discrimination can occur at any stage in the mortgage-seeking process, consideration of qualifying loans that succeed through the entire process provides a comprehensive picture of racial/ethnic inequality (compared, for instance, to only analyzing approval only at some initial stage). Table 2 reports mortgage origination rates for 2004 and 2010, along with mean distributions for our explanatory measures at individual and neighborhood levels. Although far fewer applications were submitted in 2010 compared to 2004, the percentage of mortgages that were originated across the two time points is essentially identical (i.e., 75%).

**Table 2:** Descriptions and means (standard deviations) and for loan origination, individual background characteristics, and spatial concentration and neighborhood change indicators for 2004 and 2010.

	Variable Description	2004	2010
**Loan Origination**	Whether mortgage loan application was approved and fully funded	.752 (.432)	.752 (.432)
**Applicant Attributes**			
Race/Ethnicity (ref: White)			
African American	Applicant is African American	.136 (.342)	.059 (.235)
Hispanic	Applicant is Hispanic	.130 (.337)	.071 (.257)
Asian	Applicant is Asian / Asian American	.059 (.235)	.089 (.285)
Gender (1 = Female; 0 = Male)	Applicant is Female	.329 (.470)	.291 (.454)
Owner Occupied	Dwelling will be owner occupied, not rental	.914 (.280)	.934 (.248)
Non-Government-Backed	Loan request is through a private lender	.957 (.206)	.801 (.400)
Loan Type: (ref: purchase)			
Improvement	Loan request is for home improvement	.084 (.277)	.047 (.212)
Refinance	Loan request is for refinancing not purchase	.546 (.498)	.650 (.477)
Manufactured Dwelling	Dwelling is manufactured housing	.012 (.111)	.010 (.097)
Applicant Income (1000s)	Adjusted income in thousands of dollars	85.818 (122.189)	116.177 (158.837)
Applicant Loan-to-Income	Loan-to-Income ratio for Applicant	10.410 (40.047)	2.588 (6.814)
**Neighborhood**			
Percent African American	Percent African American in neighborhood	14.113 (23.547)	9.183 (15.766)
Percent Hispanic	Percent Hispanic in neighborhood	13.879 (19.200)	12.514 (16.314)
Asian	Percent Asian in neighborhood	5.580 (9.041)	8.166 (11.697)
Change in Number of African Americans	Change in number of African Americans in neighborhood (1990–2000 interpolated for 2004; 2000–2010 for 2010 analyses)	.150 (.446)	.056 (.182)
Change in Number of Hispanics	Change in number of Hispanics in neighborhood (1990–2000 for 2004; 2000–2010 for 2010 analyses)	.265 (.507)	.042 (.212)
Change in Number of Asians	Change in number of Asians in neighborhood (1990–2000 for 2004; 2000–2010 for 2010 analyses)	.117 (.251)	.068 (.177)
Foreign Born Linguistic Isolation Unemployed Female Headed Household Married College Educated Owner Occupied Home Built Before 1970 Median Home Value Multifamily Home Creditworthiness	Percent foreign born in neighborhood Percent linguistically isolated in neighborhood Percent unemployed in neighborhood Percent the households with children headed a single female Percent married households Percent college educated Percent owner occupied Percent homes built before 1970 Median home value in dollars Percent multifamily Average FICO scores (100s)	13.34 (13.13) 4.52 (6.67) 5.50 (4.20) 12.57 (8.87) 54.42 (11.90) 26.99 (17.75) 66.30 (20.33) 50.76 (31.18) 163,602 (113,965) 24.21 (23.49) 6.925 (.127)	13.54 (12.28) 3.69 (5.66) 6.72 (3.67) 10.13 (6.64) 53.98 (12.05) 38.69 (19.93) 67.37 (18.90) 59.97 (27.94) 351,853 (221,172) 22.32 (22.70) 6.950 (.126)
N Neighborhoods N Applicants	Number of neighborhoods / 10% random sample Number of applications / 10% random sample	31,479 964,079	28,566 379,482

Our analyses build on but also extend prior analyses, noted earlier—prior analyses that have been either limited to both private-sector mortgage origination and/or the purchase of owner-occupied, single-family homes, both of which probably underestimates the extent of race/ethnic inequality given that mortgage loan origination can also be government-backed and may include rental properties, second mortgages, or mortgage refinancing. We focus on racial/ethnic inequalities in origination generally, but with recognition and important controls for type of origination and across temporal contexts (pre- and post-recession) where significant shifts in access and mortgage types occurred (see earlier Table 1 and Fig 1).

### Race/Ethnicity and other individual background attributes

Applicant characteristics were obtained from the HMDA files retrieved from the Consumer Financial Protection Bureau [106]. Race/Ethnicity is measured as a set of dummy variables indicating whether the primary borrower is African American, Hispanic, or Asian (referent = White). For 2004, overall representation of African Americans is 13.6 percent, Hispanics is 13 percent, Asians is 5.9 percent and Non-Hispanic Whites is 67.6 percent. In 2010, representation of African Americans is 9.2 percent, Hispanics is 12.5 percent, Asians is 8.2 percent and Whites is 69.2 percent. Any applications missing borrower race/ethnicity were dropped from inclusion, and applications from Native American home seekers were excluded owing to very small sample sizes. Unfortunately, since co-borrower information is absent or missing to a significant enough degree in these data, we cannot include co-borrower race in our analyses.

Beyond highlighting racial/ethnic disparities, our modeling also accounts for gender, whether the residence will be owner occupied, whether the loan would be government guaranteed, the loan purpose, manufactured home status, applicant income, and applicant debt-to-income ratio utilizing HMDA data. U.S. Manufactured homes may be referred to as “mobile homes” or “trailers” but are a specific type of factory-built housing, constructed in accordance with the U.S. Department of Housing and Urban Development’s Manufactured Home Construction and Safety Standards code after June 1976. Manufactured homes are treated distinctly as dwellings versus motor vehicles for legal and financing purposes [111]. Manufactured is dichotomous, with site-built, non-commercial one-to-four family properties as the referent. Female is a binary indicator capturing the gender of the primary borrower. Owner occupied is likewise measured in binary fashion, indicating whether the mortgage application is for the applicant’s primary residence, as opposed to a vacation or second home. Government-guaranteed includes any mortgage backed by the federal government such as Federal Housing Authority or the U.S. Department of Veterans Affairs. Loan purpose indicates what the mortgage is to be used for, including first-lien, home equity line of credit, and refinance loans. Applicant income is in thousands of dollars, while loan-to-income ratio is calculated as the value of the mortgage relative to the applicant’s income. Income is adjusted for inflation using the Consumer Price Index.

### Neighborhood racial/ethnic composition and compositional change

Consistent with our earlier discussion, we also consider within our analyses neighborhood racial/ethnic composition and neighborhood change derived from census records. Composition is relatively straightforward, indicated in our data by the percent of African American, Hispanic, Asian, and White individuals in the census tract for 2000 (for pre-recession models) and 2010 (for post-recession models) (see Table 2).

Capturing neighborhood change using percentages has important limitations. Take, for example, a neighborhood in which the White percentage of the population decreased [112]. In such a context, the minority population percentage increases by default even if the same number of minorities resided in the neighborhood during the period. A useful alternative, which we employ, is to tabulate the actual counts of each racial group from T_1_ and compare them to the counts of each group at T_2_ [112]. There are two reasons why this strategy is useful. First, individuals are more likely to view racial/ethnic presence in terms of the overall size of minority groups (i.e., “in 2004 I had one black family on my street; now there are four black families”). Secondly, the count of each group is not mechanically related to the static group percentage since the difference in each group from T_1_ to T_2_ does not essentially impact their static percentage within the neighborhood. For instance, a neighborhood experiencing a large growth in the number of Hispanics does not necessarily imply a growth in the percentage of the population that is Hispanic because this growth may have been offset by a relatively larger growth in another racial group. As a result, the correlation between proportional changes and static racial group composition should be low, and essentially lower than the correlation between static minority composition and percentage change of minority composition [112]. Using the change in group counts overcomes the statistical complications of quantifying change as the difference in percentage change and allows for an assessment of the independent effects of both racial/ethnic change and static group composition [112].

### Creditworthiness

We recognize that access to individual credit scores is challenging to obtain, so we supplement our analysis with a proxy for creditworthiness of the applicant, computed at the MSA level by race. One notable exception that includes credit score is [40], which entailed multiyear data linkages between HMDA records and private data from ATTOM and Black Knight Financial Services. In contrast to our study, the authors [40] limited their analysis to first- lien, fixed-rate, owner-occupied 30-year single-family residential loans, securitized by the Government Sponsored Enterprises or insured by the Federal Housing Administration. Of note, the authors were in receipt of funding that permitted the purchase of privately held data that could otherwise present as cost prohibitive.

We utilized MSA level FICO scores of borrowers, by race, of over 10.2 million residential mortgage loans originated from 2000 to 2009 available in public loan-level filings of government sponsored enterprise and private label mortgage-backed securities [52]. Creditworthiness is derived from compiled individual records, standardized, and concatenated from the following data sources: (GSE) Freddie Mac, Fannie Mae; (PLS) Securities & Exchange Commission, Wells Fargo Corporate Trust Services, Citigroup Structured Finance, CitiMortgage MBS, and U.S. Bank Trust Investor Reporting [113–115] and made available to the authors as part of earlier research [52]. Correlational tests using this proxy produce disparities by race/ethnicity like those witnessed at the applicant level and, thus, it is a useful control. Prior work has established the utility of such an indicator [94,116,117]. Moreover, this credit proxy helps account for omitted variable bias given the empirically demonstrated relationship between neighborhood racial/ethnic composition and place-level credit ratings [17,52].

### Neighborhood characteristics

We control for several neighborhood characteristics found in prior studies [38,46,47,69] to contribute to generally accepted measures of neighborhood quality and correspond to lender decisions surrounding whether or not to approve mortgage investment in that community. Neighborhood characteristics are sourced from HMDA files for the neighborhood in which the applicant is seeking financing and sourced from ACS and census files at the tract level, corresponding the year the application is made. Variables related to the housing stock include percentage of housing that is built before 1970, the median home value, percentage of homes that are owner occupied, and the percentage of housing that is multifamily. Vacancy rate and poverty rate were tested for inclusion but omitted due to collinearity using variance inflation factor testing. Additional Census variables were tested for inclusion but were not significant and did not improve model fit. A full list of variables considered are available from the authors upon request.

### Analytic strategy

Our analyses proceed in two steps. First, we model baseline racial/ethnic inequalities in loan origination pre- (2004) and post-recession (2010) using linear techniques. We include a second equation for each period wherein other individual attributes (e.g., gender, loan type, applicant income, and loan-to-income ratios, etc.) are introduced. Modeling in this manner highlights both baseline racial/ethnic inequalities and the degree to which they might be partially accounted for by group variations in loan type, socioeconomic background, and other individual-level predictors.

Although loan origination is itself bimodal, our analytic use of linear regression follows recent suggestions in the literature that point to potential drawbacks of nonlinear probability models such as logistic or probit for multistep modeling or group comparison [118]. Supplementary logistic regression modeling, reported in our appendices, nevertheless reveal similar inequalities to those we report in our main findings. We draw from these supplementary analyses to generate more easily interpretable and group-specific probabilities of loan origination by race/ethnicity and across periods and present these in visual form within our discussion of results.

The second step of our analyses employs hierarchical linear modeling to analyze baseline effects of neighborhood composition, compositional change, and their interactions with applicant race/ethnicity across pre- and post-recession periods. Such multilevel models are now standard in analyses of neighborhood effects [119–121]. They depart from typical regression models in assuming that each neighborhood has a different intercept coefficient β_0j_ and different slope coefficients β_1j_, β_2j_, and so on. Residual errors in multilevel models e_ij_ are estimated to have a mean of zero and a variance to be measured, and most multilevel modeling assumes the variance of the residuals is constant across all neighborhoods. Because the intercept and slope coefficients are random and vary across neighborhoods, they can be referred to as random coefficients [122]. It is assumed there is a random sample of *J* neighborhoods from the population where *J* is a large number, from which the relationship within any neighborhood *j* can be described. The intercept and slope are subscripted by *j*, allowing each neighborhood to have a unique intercept or slope. The intercept, B_0*j*_ is defined as the expected origination of an applicant who is non-Hispanic White in *j* neighborhood. The slope B_1*j*_ is the expected change in origination associated with a unit change in race and ethnicity, assuming a categorical race and ethnicity variable. The error term r_*ij*_ represents a unique effect associated with applicant *i* in *j* neighborhood. Generally, it is assumed that r_*ij*_ is normally distributed with a mean of zero and variance Ơ^2^ [121].

There are other advantages to our use of hierarchical modeling. First, recognizing and modeling hierarchical structure (i.e., individual borrowers nested within distinct compositional contexts) allows for variation within and between neighborhoods. Second, a hierarchical approach adjusts level-one coefficients for measurement error. Third, such analyses permit heterogeneity of regression across neighborhoods, such that parameter values are allowed to vary across neighborhoods. Hence, biases in parameter estimates which result from clustering are corrected. Fourth, this approach allows for unbalanced data across neighborhoods, such that both small and large neighborhoods are considered. Finally, approximating shared variance improves precision in standard errors and, thus, confidence in tests of significance (for elaboration on these points, see [123]). Our first equation in these regards assesses (level 2) baseline compositional and neighborhood change effects, with individual controls for race, gender, loan type, income, etc., along with an aggregate (level 2) control for neighborhood creditworthiness. We then report, in a second equation, statistically significant and final, trimmed interaction results between neighborhood composition, neighborhood change and the race/ethnicity of mortgage loan applicants for both 2004 and 2010. In this regard, and through an iterative process, we introduced each potential interaction between compositional indicators and applicant race/ethnicity one at a time. Only those that were significant remain in and are reported in the final modeling.

## Results

### Racial/Ethnic inequalities and mortgage loan origination

Model 1 of Table 3 reports baseline racial/ethnic inequalities in mortgage loan origination for African Americans, Hispanics, and Asians for 2004 and 2010. White mortgage loan seekers are the referent. Statistically significant and substantively sizeable inequalities in these regards are obvious and, if anything, increase somewhat over the two time periods. African American mortgage seekers are approximately .19 (2004) and .20 (2010) less likely overall to have loans originated compared to Whites. Hispanics, like African Americans, are disadvantaged relative to Whites across both time periods and fall somewhere between Whites and African American loan seekers. Asian mortgage seekers are slightly more advantaged than Whites in the pre-recession period but slightly disadvantaged after.

**Table 3:** Linear regression estimates (standard deviations) of mortgage loan origination by race/ethnic background of applicants and other individual applicant/application attributes, 2004 and 2010.

	2004		2010	
	(1)	(2)	(1)	(2)
Applicant Race/Ethnicity				
African American	-.190(.000)***	-.182(.000)***	-.201(.003)**	-.178(.003)**
Hispanic	-.080(.000)***	-.086(.000)***	-.124(.003)**	-.118(.003)**
Asian	.009(.000)***	-.010(.000)***	-.008(.002)**	-.023(.002)**
Gender (Female)		-.027(.000)***		-.027(.002)**
Owner Occupied		-.006(.000)***		.103(.003)**
Non-Government-Backed		-.086(.000)***		.075(.002)**
Loan Type (ref: purchase)				
Improvement		-.248(.000)***		-.289(.003)**
Refinance		-.112(.000)***		-.140(.002)**
Manufactured Dwelling		-.355(.000)***		-.334(.007)**
Applicant Income (1000s)		.000(.000)***		.000(.000)***
Applicant Loan-to-Income		.000(.000)***		-.003(.000)***
Constant	.788	.959	.773	.038
Adjusted R^2^	.024	.065	.016	.052
N of applicants	964,079	964,079	379,482	379,482

*p < .05

**p < .01

***p < .001 (one-tailed tests of significance).

**Fig 2:**
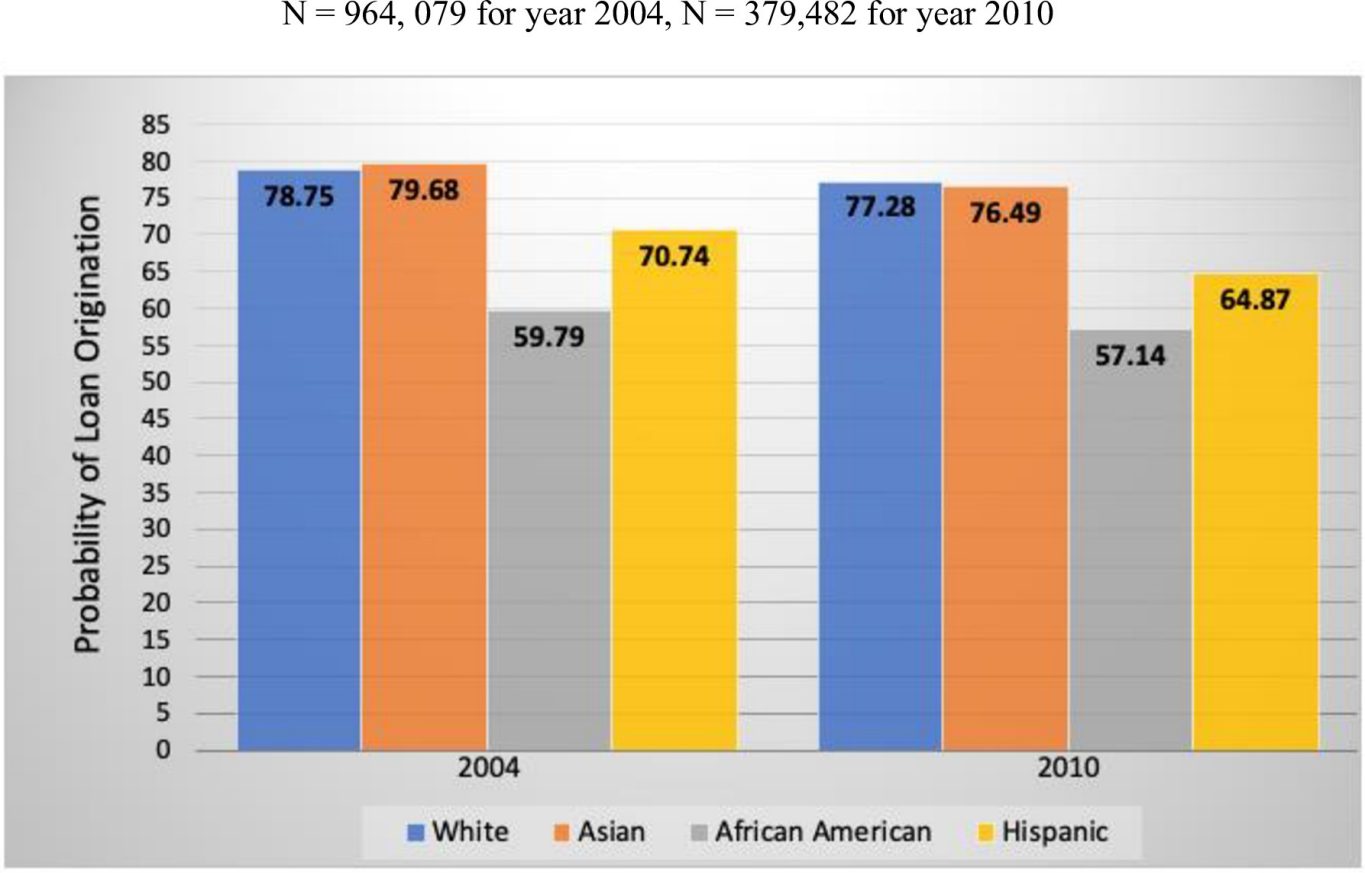
Probability of mortgage loan origination by race/ethnicity, pre- and post-recession.

Fig 2 reports these patterns in the form of loan origination probabilities by racial/ethnic group, drawing from logistic regression estimates reported S1 Table. Here we find that the overall probability of origination for White applicants ranges from 77 to 79 percent, but only 57 to 60 percent for African Americans and from 65 to 71 percent for Hispanic applicants. Such inequalities, which reflect about a 20 percent disadvantage for African Americans and around a 10 percent disadvantage for Hispanics, are substantively sizeable, noteworthy, and consistent with our first hypothesis. The differences between Whites and Asian applicants are not nearly as substantively large. Whether these observed and sizeable gaps, particularly for African Americans and Hispanics, are a product of variation in loan types, type of dwelling, etc. or partially a function of more basic background disadvantages in socioeconomic status is addressed in Model 2 of Table 3.

Coefficients reported in Model 2, along with the shifts observed between 2004 and 2010, are generally in line with what one might expect. Specifically, female applicants are disadvantaged on average compared to their male counterparts, and respondents with higher incomes are more likely to see their loans originated across both time periods. Notable shifts, however, are also witnessed in a way suggesting that lending criteria became more stringent following the housing bubble burst and recession. Particularly notable is that the effect of debt-to-income ratios reverses across time periods, suggesting that the criteria used in assessing loan application and in determining origination became increasingly rigid.

Especially interesting, at least relative to our focus on race/ethnic-specific inequalities and somewhat countervailing expectations (hypotheses 2 and 3), is the fact that the inclusion of other background attributes barely moves the inequality needle. In fact, comparing coefficient changes across Models 1 and 2 and by periods, we find only minor reductions in loan origination inequalities observed for African Americans and little to no reduction for Hispanics. In fact, for African American loan seekers, the inequality gap is reduced by only about 4 percent in 2004 and 11 percent in 2010 once background characteristics are taken into account. Thus, even though more stringent algorithmic criteria perhaps slightly reduced race-specific inequalities for African Americans across the two time periods (hypothesis 2), significant and sizeable disparities persist for African Americans and Hispanics seeking and securing home loans (hypothesis 3).

Above and beyond rigid racial/ethnic inequalities in loan origination across time, it is important to recognize that the sizeable drop in mortgage applications post-recession—a drop of approximately 60 percent—is likewise not equally distributed. African American and Hispanic rates of application, in fact, disproportionately plummeted during the post-recession period to only 17 and 21 percent of what they were prior to the recession. This means that the inequalities about which we are speaking are two-fold: (1) prospective African and Hispanic home buyers are consistently disadvantaged across time in the loan seeking and approval process; and (2) a disparate portion of African American and Hispanic home seekers were removed from mortgage seeking and home purchasing altogether during the post-recession period.

Whether mortgage loan origination and the inequalities reported thus far are shaped by the racial/ethnic character of the area in which the loan is requested is an important sociological question in its own right [4,124]. We address it next through hierarchical modeling of inequalities in loan origination, race/ethnicity of applicants, and the compositional and representational changes of the places within which loans were requested.

### Compositional dynamics, loan origination, race/ethnic intersections

Model 1 of Table 4 reports hierarchical linear modeling estimates of the inequalities already highlighted, but now with baseline effects of neighborhood racial/ethnic composition and neighborhood race/ethnic change. Also included are level-1 (individual) controls reported earlier and a level-2 control for neighborhood credit worthiness. Modeling in this way helps adjust for clustering effects and acknowledges the ongoing and contemporary pertinence of racial/ethnic segregation across the U.S. Model 2 takes the analyses a step further and reports trimmed interactional modeling between compositional indicators and the race/ethnicity of applicants.

**Table 4:** Hierarchical linear modeling estimates (standard deviations) of mortgage loan origination by neighborhood racial/ethnic composition, compositional change, and individual applicant attributes (Model 1), and trimmed interaction modeling (Model 2) of applicant race and composition/compositional change, 2004 and 2010.

	2004		2010	
	(1)	(2)	(1)	(2)
**Neighborhood Minority Composition**				
Percent African American	-.001(.000)***	-.002(.000)***	-.002(.000)***	-.002(.000)***
Percent AA x African American Applicant		.000(.000)***		ns
Percent Hispanic	-.001(.000)***	-.001(.000)***	-.001(.000)***	-.001(.000)***
Percent Hispanic x Hispanic Applicant		ns		.000(000)*
Percent Asian	.002 (0.000)***	.002(.000)***	.001(.000)***	.001(.000)***
Percent Asian x Asian Applicant		ns		ns
**Compositional Change**				
Increasing Number of African Americans	.003(.003)	-.003(.000)	.013(.005)**	.007(.005)
Increasing AA x African American Applicant		.013(.002)***		.023(.009)*
Increasing Number of Hispanics	-.005(.003)	-.009(.003)**	.002(.004)	-.004(.004)
Increasing Hispanic x Hispanic Applicant		.010(.002)***		.024(.008)**
Increasing Number of Asians	.001(.006)	.003(.006)	.013(.005)*	.013(.005)*
Increasing Asian X Asian Applicant		ns		Ns
Applicant Race/Ethnicity				
African American	-.124(.002)***	-.139(.002)***	-.115(.004)**	-.118(.005)***
Hispanic	-.064(.002)***	-.069(.002)***	-.080(.003)**	-.082(.003)***
Asian	-.032(.002)***	-.032(.002)***	-.036(.003)***	-.036(.003)***
Gender (Female)	-.023(.001)***	-.023(.001)***	-.022(.002)***	-.022(.002)***
Owner Occupied	-.038(.002)***	-.039(.002)**	.087(.003)***	.087(.003)***
Non-Government-Backed	-.121(.002)***	-.121(.002)**	.065(.002)***	.065(.002)***
Loan Type (ref: purchase)				
Improvement	-.238(.002)***	-.237(.002)**	-.282(.003)***	-.282(.003)***
Refinance	-.113(.001)***	-.113(.001)***	-.145(.002)***	-.145(.002)***
Manufactured Dwelling	-.320(.004)***	-.320(.000)**	-.329(.007)***	-.329(.007)***
Applicant Income (1000s)	.000(.000)***	.000(.000)***	.000(.000)***	.000(.000)***
Applicant Loan-to-Income Ratio	.000(.000)***	.000(.000)***	-.003(.000)***	-.003(.000)***
Creditworthiness	.087(.009)***	.086(.009)**	.018(.007)**	.018(.007)*
Constant	.429	.442	.647	.648
N of Neighborhoods N of Applicants	31,479 964,079	31,479 964,079	28,566 379,482	28,566 379,482

*p < .05

**p < .01

***p < .001 (one-tailed tests of significance).

Noteworthy from the outset is that the inclusion of neighborhood composition and change indicators in Model 1 helps explain African American and Hispanic disadvantages in loan origination. In fact, when we compare the inequalities reported earlier in Model 2 of Table 3 to those reported in Model 1 of Table 4, when composition neighborhood change is included, we see that between one-third and one-half of the inequalities that African Americans and Hispanics experience in mortgage loan origination are now taken into account. Acknowledging this helps make explicit the ongoing significance of racial segregation and, just as important, the willingness (or lack thereof) of lending institutions to invest in the neighborhoods where prospective minority home buyers are most likely to request loans.

Although we find limited evidence in Model 1 that changes in racial/ethnic representation in neighborhoods over the two time periods matter, lender aversion to loan origination in highly concentrated African American and Hispanic neighborhoods is obvious in these models and is consistent with our fourth hypothesis. The exception has to do with what seems to be increased lender investment in areas with growing African American and Asian populations in 2010. As noted momentarily, however, and according to our interaction modeling, any such spatial change effects (i.e., minority population growth) only seem to be only consequential when the loan applicant is African American or Hispanic. To the extent this is the case, and that applicant race and minority population growth align in systematic ways when it comes to lending, it reflects a pattern of biased lending that will tend to reify patterns of racial residential segregation. Such effects barely change and persist to a high degree into the post-recession period. Higher African American and Hispanic neighborhood concentrations are associated with a diminished likelihood of mortgage loan origination in general, while higher Asian concentration has the reverse, positive association. For both 2004 and 2010, the average creditworthiness of those in a given neighborhood—something lending institutions equate with overall risk—likewise matters in an expected and positive direction. Notably, however, neighborhood creditworthiness does not negate either the racial/ethnic inequalities we have reported or the observed effects of neighborhood racial/ethnic composition.

Model 2 of Table 4 reports final and trimmed interaction tests between our compositional and neighborhood change indicators and applicant race/ethnicity. The positive interaction between applicant race (i.e., African American) and Black population concentration in 2004, as well as between African American and Hispanic borrowers and African American and Hispanic neighborhood minority population growth in both 2004 and 2010, suggests that lenders, explicitly or implicitly, continue to gauge racial fit/appropriateness of loan requests. The interaction between African American applicants and overall Black concentration becomes non-significant in 2010, perhaps pointing to a more explicit disconnect on lenders’ part between race/ethnicity of applicants and neighborhood, although connections with African American and Hispanic neighborhood population growth persist into the post-recession period. Interaction coefficients pertaining to minority population growth specifically suggest that baseline disadvantages in African American and Hispanic mortgage loan originations dissipate and are reduced, at least somewhat, when the loan request corresponds to a neighborhood that has been/is becoming more African American or Hispanic, respectively, over time.

Such effects, especially when considered alongside the persistent significance of individual race/ethnicity, greater loan restrictions over time, and the fact that baseline African American and Hispanic population concentrations generally reduce the overall likelihood of loan origination even into the post-recession period, point to several problematic pathways by which racial/ethnic inequality in mortgage lending continues to matter. These pathways include: (1) the disparate selection of African-American and Hispanic homebuyers out of mortgage seeking and homebuying when criteria for approval becomes especially rigid (as noted by loan origination shifts between pre- and post-recession periods; Fig 1); (2) ongoing and direct lending biases against prospective African American and Hispanic homebuyers (Table 3 and Fig 2); (3) general aversions to the infusion of capital into more highly concentrated African-American and Hispanic concentrated neighborhoods (Table 4, Model 1); and (4) a reification of residential segregation when lending practices tend to boost approval when applicant race/ethnicity corresponds to specific neighborhood racial/ethnic over-time changes (Table 4, Model 2).

## Discussion

The last several decades of sociological research have made clear that housing carries a foundational connection to intergenerational inequality and wealth accumulation [5,66,125,126]. Significant racial/ethnic inequalities persist in these regards despite a half-century of civil rights law and anti-discrimination legislation. In this article, we address such enduring gaps as they pertain specifically to the mortgage loan approval process, whether observed inequalities shifted in any significant way following the great recession and the housing boom and bust, and the degree to which spatial dynamics and the segregated character of neighborhoods continue to play a role.

Our analyses and findings, drawing on over a million randomly selected mortgage loan applications matched with American Communities Survey locality data for 2004 and 2010, point to persistent and contemporary racial/ethnic inequalities in the mortgage loan approval process, particularly for African American and Hispanic home seekers—inequalities that have barely changed across time. The likelihood of African American and Hispanic mortgage loan origination remains significantly lower than that for White and Asian applicants, and these patterns are barely accounted for by individual-level factors like income, debt-to-income ratio, mortgage loan type, etc. This finding, in our view, underscores the potential relevance of contemporary discrimination, whether enacted informally by lending agencies and mortgage brokers or more structurally and implicitly through financial algorithms [39,67]. Automated underwriting—including the reliance on algorithms and machine learning—is a fundamental issue, in our view, that future research should address more directly and explicitly given the possibility that such practices may support racial capitalism [127–130].

Although our finding may seem at first glance to run counter to other important and recent work [50] that suggests some movement toward greater racial/ethnic equity in the post-recession period, it is important to recognize that our work differs in its inclusion of government-backed loans, home improvement and refinance loans, and the purchase of manufactured dwellings. The inclusion of these alternative mortgage types alongside private and first-time home purchases is especially important when one considers greater government involvement in the post-recession period (Table 1), some shifts across periods in the types of loans approved (Fig 1), and the fact that greater scrutiny often benefits the already advantaged (e.g., refinancing an existing mortgage at lower interest rates). It is for these reasons that we hope future analyses will remain attuned to institutional practices that not only potentially exclude disadvantaged groups but also those that allow for opportunity hoarding among the already privileged.

Beyond the group-level racial/ethnic inequalities highlighted, our analyses also made use of hierarchical modeling techniques in order to interrogate potential spatial sources of inequality tied to segregation [6] and the extent to which conditional associations between neighborhood composition, change, and applicant race/ethnicity exist. Our findings in these regards clearly point to an enduring impact of spatial segregation and the general devaluation of minority concentrated neighborhoods. Specifically, the likelihood of mortgage loan origination is significantly depressed in higher concentrated African American and Hispanic neighborhoods, and such effects remain consistent during both pre- and post-recession periods. It is more than plausible that effects are at least partially driven by standard algorithmic neighborhood valuation formulas—formulas that appear neutral in their use of aggregate indicators (e.g., crime rates, education levels, average property values, etc.) but that depress loan approval and generally undercut investment in minority neighborhoods. One interesting caveat to this pattern is revealed by conditional associations between applicant race/ethnicity and neighborhood change over the last decade. Here, a key finding is that origination in highly concentrated minority neighborhoods increases for Black and Hispanic homebuyers, at least minimally, when the applicant’s race/ethnicity corresponds to the racial/ethnic direction a given neighborhood has been moving, but for Black and Hispanic homebuyers is negatively associated with overall racial concentration. Future research could delve into the protective facets of the racially changing neighborhood, which if uncovered, could be capitalized on for improving origination rates in racially unchanged minority neighborhoods for Black and Hispanic buyers.

Like most analyses using aggregate quantitative data, ours admittedly has limitations—limitations that we hope future research and data collection efforts can address. Our decision to utilize a 10% random sample for the top 100 metropolitan regions was not a new strategy but not employed with most mortgage studies, especially those focused on smaller metros or one location. Although sensitive checks suggest our randomly selected sample is comparable to other random samples, it is possible our sample is not perfectly representative of the top 100 metropolitan areas, so this is a limitation. As noted earlier, the applicant data at our disposal does not include robust information on cosigner race/ethnicity and was consequently excluded. It thus remains unclear whether interracial- and inter-ethnic couples experience less differential treatment than exclusively African American or Hispanic couples, or whether the reality of being an interracial couple, in and of itself, generates unique exclusions in the housing market. Without more detailed co-signer information, future research might consider deeper immersion into housing discrimination case files to get at this [44]. Qualitative case materials can provide clearer articulation of what occurred and who was affected, while also allowing for simultaneous assessment of regional and local institutional processes—processes that are important for the historical and contemporary reification of racial/ethnic inequality across geographic space and place-based resource allocation, in this regard, see for instance [131]. In addition to co-applicant, there are other variables at the applicant, neighborhood, and metropolitan levels that we did not include for reasons of collinearity or lack of significance. Their exclusion might be seen as a limitation. On the other hand, more serious interrogation of other variables could very well open up avenues for future inquiry (e.g., jumbo loan status, preapproval request, area job market variables.

Despite such analytic caveats, we believe that the substantive and broader sociological implications of our findings should be taken seriously and viewed as complementary and informative to several literatures and sub-specialties within the field. Most directly, our arguments and results speak to ongoing sociological concerns regarding racial/ethnic inequality, the processes that reify it, and intergenerational disadvantage. Housing is integral to wealth accumulation. Indeed, home ownership is: (1) a mechanism of intergenerational transfer of privilege and status that both disproportionately benefits the White population and can easily be conceived of as part and parcel of opportunity hoarding [132]; (2) a safety net for families who experience job-related, financial, or medical shocks; and (3) a form of wealth and leverage that can be converted into other forms of investment (e.g., collateral for small business loans, borrowing to cover college costs for one’s children, borrowing for healthcare expenses etc.); (4) access to safe, low-crime neighborhoods with strong public safety infrastructure that informs health and well-being. In these regards, our findings are as much about White privilege and advantage as they are about inequalities experienced by African Americans and Hispanics.

Beyond direct associations with wealth or wealth transfers intergenerationally, inequalities in mortgage loan origination and their consequences for home ownership are also quite meaningful for other key institutional domains of interest to sociologists. This includes access to quality public education, good infrastructure and safe environments, and strong labor markets with opportunities for mobility in employment, and access to food and well-resourced health services. Housing and home ownership are especially central to such topics. We correspondingly hope the future scholarship will extend upon our analyses using a variety of methods and in a manner where links to population well-being, mobility, and opportunity are made even more explicit. Doing so would provide not only important insights regarding meaningful sociological processes and mechanisms but also directions, recommendations, and justifications for more effective civil rights oversight and housing policy in the future.

## Supporting information

S1 Table Log-odds estimates (standard deviations) of loan origination by race/ethnicity, 2004 and 2010. (PDF)

## References

[1] Du Bois WEB. The Souls of Black Folk. State College, P.A.: Pennsylvania State University; 2006.

[2] Massey DS, Denton NA. American Apartheid: Segregation and the Making of the Underclass. Cambridge: Harvard University Press; 1993.

[3] Oliver ML, Shapiro TM. Black Wealth/White Wealth: A New Perspective on Racial Inequality. New York: Routledge; 1995.

[4] Thomas ME, Moye R, Henderson L, Horton HD. Separate and Unequal: The Impact of Socioeconomic Status, Segregation, and the Great Recession on Racial Disparities in Housing Values. Sociology of Race and Ethnicity. 2018 Apr;4(2):229–44.

[5] Fligstein N, Rucks-Ahidiana Z. The Rich Got Richer: The Effects of the Financial Crisis on Household Well-Being, 2007–2009. In: Keister LA, Roscigno VJ, editors. Research in the Sociology of Work [Internet]. Emerald Group Publishing Limited; 2016 [cited 2024 Mar 29]. p. 155–85. Available from: https://www.emerald.com/insight/content/doi/ doi: 10.1108/S0277-283320160000028011/full/html

[6] Lipsitz G. How Racism Takes Place. Philadelphia, PA: Temple University Press; 2011.

[7] O’Neil Kuebler M. Closing the Wealth Gap: A Review of Racial and Ethnic Inequalities in Homeownership. Sociology Compass. 2013 Aug;7(8):670–85.

[8] Krivo LJ, Peterson RD, Rizzo H, Reynolds JR. Race, Segregation, and the Concentration of Disadvantage: 1980–1990. Social Problems. 1998 Feb;45(1):61–80.

[9] Krivo LJ, Peterson RD, Kuhl DC. Segregation, Racial Structure, and Neighborhood Violent Crime. American Journal of Sociology. 2009 May;114(6):1765–802. doi: 10.1086/597285 19852253

[10] Wilson WJ. The Truly Disadvantaged: The Inner City, The Underclass, and Public Policy. Chicago: University of Chicago Press; 2012.

[11] Dietz RD, Haurin DR. The social and private micro-level consequences of homeownership. Journal of Urban Economics. 2003 Nov;54(3):401–50.

[12] Rasmussen DW, Megbolugbe IF, Morgan BA. Using the 1990 Public Use Microdata Sample to Estimate Potential Demand for Reverse Mortgage Products. Journal of Housing Research. 1995;1–23.

[13] Racial Finnigan R. and ethnic stratification in the relationship between homeownership and self-rated health. Social Science & Medicine. 2014 Aug;115:72–81. 24953499 10.1016/j.socscimed.2014.06.019 PMC4301401

[14] Ortiz SE, Zimmerman FJ. Race/Ethnicity and the Relationship Between Homeownership and Health. Am J Public Health. 2013 Apr;103(4):e122–9. 10.2105/AJPH.2012.300944 PMC3673264 23409877

[15] Park GR, Park H, Kim J. Homeownership and Psychological Resources Among Older Adults: Do Gender and Mortgage Status Moderate Homeownership Effects? J Aging Health. 2022 Jan;34(1):88–99. 10.1177/08982643211029174 34192959

[16] Bond C, Williams R. Residential Segregation and the Transformation of Home Mortgage Lending. Social Forces. 2007 Dec 1;86(2):671–98.

[17] Rugh JS, Massey DS. Racial Segregation and the American Foreclosure Crisis. Am Sociol Rev. 2010 Oct;75(5):629–51. 10.1177/0003122410380868 PMC4193596 25308973

[18] Williams R, Nesiba R, Mcconnell ED. The Changing Face of Inequality in Home Mortgage Lending. Social Problems. 2005 May;52(2):181–208.

[19] Hill RP, Kozup JC. Consumer Experiences with Predatory Lending Practices. Journal of Consumer Affairs. 2007 Jun;41(1):29–46.

[20] Immergluck D. The accumulation of lender-owned homes during the US mortgage crisis: examining metropolitan REO inventories. Housing Policy Debate. 2010 Sep;20(4):619–45.

[21] Farley R. The Waning of American Apartheid? Contexts. 2011 Aug;10(3):36–43.

[22] Ray V. A Theory of Racialized Organizations. Am Sociol Rev. 2019 Feb;84(1):26–53.

[23] Tomaskovic-Devey D, Avent-Holt D. Relational inequalities: An organizational approach. Oxford University Press; 2019.

[24] Pager D. The Mark of a Criminal Record. American Journal of Sociology. 2003 Mar;108(5):937–75.

[25] Roscigno VJ. The face of discrimination: How race and gender impact work and home lives. Rowman & Littlefield Publishers; 2007.

[26] Supremacy Bonilla-Silva E. and Racism in the Post-Civil Rights Era. Boulder: Lynne Rienner Publishers; 2001.

[27] Feagin J. Systemic Racism: A Theory of Oppression. Milton Park, UK: Routledge; 2013.

[28] Robinson JN. Making Markets on the Margins: Housing Finance Agencies and the Racial Politics of Credit Expansion. American Journal of Sociology. 2020 Jan 1;125(4):974–1029.

[29] Yinger J. Closed Doors, Opportunities Lost: The Continuing Costs of Housing Discrimination. New York: Russell Sage Foundation; 1995.

[30] Besbris M, Faber JW. Investigating the Relationship Between Real Estate Agents, Segregation, and House Prices: Steering and Upselling in New York State. Sociological Forum. 2017 Dec;32(4):850–73.

[31] Pager D, Shepherd H. The Sociology of Discrimination: Racial Discrimination in Employment, Housing, Credit, and Consumer Markets. Annu Rev Sociol. 2008 Aug 1;34(1):181–209. 10.1146/annurev.soc.33.040406.131740 PMC2915460 20689680

[32] Korver-Glenn E. Race Brokers: Housing Markets and Segregation in 21st Century Urban America. New York: Oxford University Press; 2021.

[33] Turner MA, Santos R, Levy DK, Wissoker DA, Aranda C, Pitingolo R. Vol. 2012 full report. Washington, DC: Urban Institute; 2013.

[34] Ross SL, Turner MA. Housing Discrimination in Metropolitan America: Explaining Changes between 1989 and 2000. Social Problems. 2005 May;52(2):152–80.

[35] Wilson WJ. The geography of opportunity: Race and housing choice in metropolitan America. Rowman & Littlefield; 2006. (Edited by Xavier De Souza Briggs).

[36] Taylor KY. Race for profit: How banks and the real estate industry undermined black homeownership. Chapel Hill: UNC Press Books; 2019.

[37] Feinberg RM, Nickerson D. Crime and residential mortgage default: an empirical analysis. Applied Economics Letters. 2002 Mar;9(4):217–20.

[38] O’Neil MM. Housing Policy, Race, Inequality, and Disparate Impact. Phylon. 2018;Remembering W.E.B. Du Bois and Martin Luther King, Jr. 51(1):60–92.

[39] Haupert T. The Racial Landscape of Fintech Mortgage Lending. Housing Policy Debate. 2022 Mar 4;32(2):337–68.

[40] Bartlett R, Morse A, Stanton R, Wallace N. Consumer-lending discrimination in the FinTech Era. Journal of Financial Economics. 2022 Jan;143(1):30–56.

[41] Fuster A, Plosser M, Schnabl P, Vickery J. The Role of Technology in Mortgage Lending. The Review of Financial Studies. 2019 May 1;32(5):1854–99.

[42] Bianchi SM, Farley R, Spain D. Racial Inequalities in Housing: An Examination of Recent Trends. Demography. 1982 Feb 1;19(1):37–51. 7067869

[43] Flippen C. Unequal Returns to Housing Investments? A Study of Real Housing Appreciation among Black, White, and Hispanic Households. Social Forces. 2004 Jun 1;82(4):1523–51.

[44] Roscigno VJ, Karafin DL, Tester G. The Complexities and Processes of Racial Housing Discrimination. Social Problems. 2009 Feb;56(1):49–69.

[45] Faber JW. Racial Dynamics of Subprime Mortgage Lending at the Peak. Housing Policy Debate. 2013 Apr;23(2):328–49.

[46] Faber JW. Segregation and the Geography of Creditworthiness: Racial Inequality in a Recovered Mortgage Market. Housing Policy Debate. 2018 Mar 4;28(2):215–47.

[47] Haupert T. Racial Patterns in Mortgage Lending Outcomes During and After the Subprime Boom. Housing Policy Debate. 2019 Nov 2;29(6):947–76.

[48] Wyly E, Ponder CS. Gender, age, and race in subprime America. Housing Policy Debate. 2011 Sep;21(4):529–64.

[49] Immergluck D. Neighborhoods in the Wake of the Debacle: Intrametropolitan Patterns of Foreclosed Properties. Urban Affairs Review. 2010 Sep;46(1):3–36.

[50] Loya J, Flippen C. The Great Recession and Ethno-Racial Disparities in Access to Mortgage Credit. Social Problems. 2021 Oct 19;68(4):1026–50.

[51] Boehm TP, Thistle PD, Schlottmann A. Rates and race: An analysis of racial disparities in mortgage rates. Housing Policy Debate. 2006 Jan;17(1):109–49.

[52] O’Neil Kuebler MM, Rugh JS. New evidence on racial and ethnic disparities in homeownership in the United States from 2001 to 2010. Social Science Research. 2013 Sep;42(5):1357–74. doi: 10.1016/j.ssresearch.2013.06.004 23859736

[53] Charles KK, Hurst E. The Transition to Home Ownership and the Black-White Wealth Gap. Review of Economics and Statistics. 2002 May;84(2):281–97.

[54] Santiago AM, Galster GC, Santiago-San Roman AH, Tucker CM, Kaiser AA, Grace RA. Foreclosing on the American dream? The financial consequences of low-income homeownership. Housing Policy Debate. 2010 Sep;20(4):707–42.

[55] Pattillo McCoy M. Black Picket Fences: Privilege and Peril among the Black Middle Class. Chicago, IL: University of Chicago Press; 1999.

[56] Spader JS, Quercia RG. Mobility and exit from homeownership: Implications for community reinvestment lending. Housing Policy Debate. 2008 Jan;19(4):675–709.

[57] Boehm TP, Schlottmann AM. The dynamics of race, income, and homeownership. Journal of Urban Economics. 2004 Jan;55(1):113–30.

[58] Haurin DR, Rosenthal SS. The sustainability of homeownership: Factors affecting the duration of homeownership and rental spells. US Department of Housing and Urban Development: Office of Policy Development and Research; 2004.

[59] Geography Sanchez-Moyano R. and Hispanic homeownership: a review of the literature. J Hous and the Built Environ. 2021 Mar;36(1):215–40.

[60] Massey DS, Rugh JS. The Great Recession and the Destruction of Minority Wealth. Current History. 2018 Nov 1;117(802):298–303.

[61] Barr MS. No Slack: The Financial Lives of Low-Income Americans. Washington. Washington, DC: Brookings Institution Press; 2012.

[62] Bayer P, Charles KK. Divergent Paths: A New Perspective on Earnings Differences Between Black and White Men Since 1940. The Quarterly Journal of Economics. 2018 Aug 1;133(3):1459–501.

[63] Desmond M, Western B. Poverty in America: New Directions and Debates. Annu Rev Sociol. 2018 Jul 30;44(1):305–18.

[64] Harding DJ, Morenoff JD, Wyse J. On the outside: Prisoner reentry and reintegration. University of Chicago Press; 2019.

[65] Byron RA. Discrimination, Complexity, and the Public/Private Sector Question. Work and Occupations. 2010 Nov;37(4):435–75.

[66] Conley D. Being Black, Living in the Red. Berkeley: University of California Press; 2009.

[67] Eubanks Eubanks V., V. Automating Inequality: How High-Tech Tools Profile, Police, and Punish the Poor. New York: St. Martin’s Press; 2018.

[68] Noble SU. Algorithms of oppression: How search engines reinforce racism. NYU Press; 2018. 10.1126/science.abm5861 34709921

[69] O’Neil MM. Crossing the Color Line in the 21st Century: Mortgaging Increasingly Diverse Neighborhoods. SSRN Electronic Journal [Internet]. 2019; Available from: https://ssrn.com/abstract=3415647.

[70] Schmeckpeper K, Roberts S, Ouellet M, Malencia M, Jain D, Gosrich W, et al. Algorithm Transparency through the Fair Credit Reporting Act (FCRA). Journal of Science Policy & Governance. 2021;18(4).

[71] Ruha B. Race after technology: Abolitionist tools for the new Jim code. John Wiley & Sons; 2019.

[72] Friedline T. Banking on a Revolution: Why Financial Technology Won’t Save a Broken System. Oxford: Oxford University Press; 2020.

[73] Crowder K. The Racial Context of White Mobility: An Individual-Level Assessment of the White Flight Hypothesis. Social Science Research. 2000 Jun;29(2):223–57.

[74] Crowder K, South SJ. Spatial Dynamics of White Flight: The Effects of Local and Extralocal Racial Conditions on Neighborhood Out-Migration. Am Sociol Rev. 2008 Oct;73(5):792–812. 10.1177/000312240807300505 PMC2835167 20221414

[75] Harris DR. “Property Values Drop When Blacks Move In, Because…”: Racial and Socioeconomic Determinants of Neighborhood Desirability. Am Sociol Rev. 1999 Jun;64(3):461–79.

[76] Hyra DS. The New Urban Renewal: The Economic Transformation of Harlem and Bronzeville. Chicago: The University of Chicago Press; 2008.

[77] Sampson RJ. Racial Stratification and the Durable Tangle of Neighborhood Inequality. The ANNALS of the American Academy of Political and Social Science. 2009 Jan;621(1):260–80.

[78] Wahl AMG, Breckenridge RS, Gunkel SE. Latinos, residential segregation and spatial assimilation in micropolitan areas: Exploring the American dilemma on a new frontier. Social Science Research. 2007 Sep;36(3):995–1020.

[79] Howell J, Korver-Glenn E. The Increasing Effect of Neighborhood Racial Composition on Housing Values, 1980–2015. Social Problems. 2021 Oct 19;68(4):1051–71.

[80] Wilson WJ, Taub RP. There Goes the Neighborhood: Racial, Ethnic, and Class Tensions in Four Chicago Neighborhoods and Their Meaning for America. New York: Knopf; 2006.

[81] Bruch EE, Mare RD. Neighborhood Choice and Neighborhood Change. American Journal of Sociology. 2006 Nov;112(3):667–709.

[82] Emerson MO, Chai KJ, Yancey G. Does Race Matter in Residential Segregation? Exploring the Preferences of White Americans. American Sociological Review. 2001 Dec;66(6):922.

[83] Schelling TC. Dynamic models of segregation†. The Journal of Mathematical Sociology. 1971 Jul;1(2):143–86.

[84] Taub RP, Taylor DG, Dunham JD. Paths of neighborhood change: Race and crime in urban America. Chicago: University of Chicago Press; 1984.

[85] Banzhaf HS, Walsh RP. Segregation and Tiebout sorting: The link between place-based investments and neighborhood tipping. Journal of Urban Economics. 2013 Mar;74:83–98.

[86] Brasington D, Haurin DR. Educational Outcomes and House Values: A Test of the value added Approach*. J Regional Sci. 2006 May;46(2):245–68.

[87] Downes TA, Zabel JE. The impact of school characteristics on house prices: Chicago 1987–1991. Journal of Urban Economics. 2002 Jul;52(1):1–25.

[88] Kane T, Staiger D, Riegg S. School Quality, Neighborhoods and Housing Prices: The Impacts of school Desegregation [Internet]. Cambridge, MA: National Bureau of Economic Research; 2005 May [cited 2024 Mar 29] p. w11347. Report No.: w11347. Available from: http://www.nber.org/papers/w11347.pdf.

[89] Apgar WC, Duda M, Nawrocki Gorey R. The Municipal Cost of Foreclosures: A Chicago Case Study: Homeownership Preservation Foundation. 2005. (Housing Finance Policy Research Paper).

[90] Immergluck D, Smith G. The Impact of Single-family Mortgage Foreclosures on Neighborhood Crime. Housing Studies. 2006 Nov 1;21(6):851–66.

[91] Schuetz J, Been V, Ellen IG. Neighborhood effects of concentrated mortgage foreclosures. Journal of Housing Economics. 2008 Dec;17(4):306–19.

[92] Teasdale B, Clark LM, Hinkle JC. Subprime Lending Foreclosures, Crime, and Neighborhood Disorganization: Beyond Internal Dynamics. Am J Crim Just. 2012 Jun;37(2):163–78.

[93] Bocian Gruenstein D, Li W, Quercia RG. Lost Ground: Disparities in Mortgage Lending and Foreclosures. Washington, DC: Center for Responsible Lending; 2011.

[94] Calem PS, Gillen K, Wachter S. The Neighborhood Distribution of Subprime Mortgage Lending. The Journal of Real Estate Finance and Economics. 2004 Dec;29(4):393–410.

[95] Calem PS, Hershaff JE, Wachter SM. Neighborhood Patterns of Subprime Lending: Evidence from Disparate Cities. SSRN Journal [Internet]. 2004 [cited 2024 Mar 29]; Available from: http://www.ssrn.com/abstract=583102.

[96] Moulton S. Originating lender localness and mortgage sustainability: an evaluation of delinquency and foreclosure in Indiana’s mortgage revenue bond program. Housing Policy Debate. 2010 Sep;20(4):581–617.

[97] Munnell AH, Tootell GM, Browne LE, McEneaney J. Mortgage lending in Boston: Interpreting HMDA data. The American Economic Review. 1996;25–53.

[98] Stuart G. Discriminating risk: The US mortgage lending industry in the twentieth century. Cornell University Press; 2003.

[99] Logan JR, Zhang C. Global Neighborhoods: New Pathways to Diversity and Separation. American Journal of Sociology. 2010 Jan;115(4):1069–109. doi: 10.1086/649498 24013784 PMC3763509

[100] Logan JR, Stults BJ, Farley R. Segregation of minorities in the metropolis: two decades of change. Demography. 2004 Feb 1;41(1):1–22. 10.1353/dem.2004.0007 15074122

[101] Charles CZ. The Dynamics of Racial Residential Segregation. Annu Rev Sociol. 2003 Aug;29(1):167–207.

[102] Massey DS, Denton NA. Trends in the Residential Segregation of Blacks, Hispanics, and Asians: 1970–1980. American Sociological Review. 1987 Dec;52(6):802.

[103] Wilkes R, Iceland J. Hypersegregation in the twenty-first century. Demography. 2004 Feb 1;41(1):23–36. 10.1353/dem.2004.0009 15074123

[104] Census Bureau U.S. Profile of General Population and Housing Characteristics: 2010. 2013.

[105] Howell J. The unstudied reference neighborhood: Towards a critical theory of empirical neighborhood studies. Sociology Compass. 2019 Jan;13(1):e12649.

[106] Consumer Financial Protection Bureau (CFPB). Home Mortgage Disclosure Data: download HMDA data. [Internet]. 2023. Available from: https://www.consumerfinance.gov/data-research/hmda/historic-data/.

[107] Gerardi K, Shapiro AH, Willen PS. Subprime outcomes: Risky mortgages, homeownership experiences, and foreclosures. Federal Reserve Bank of Boston. Working Papers, No. 07–15.

[108] Census Bureau U.S. Accessing Public Data. [Internet]. 2023. Available from: https://www.census.gov/about/policies/open-gov/open-data.html.

[109] Census Bureau U.S. American Community Survey Data. [Internet]. 2023. Available from: https://www.census.gov/programs-surveys/acs/data.html.

[110] Logan JR, Zhang W, Stults BJ, Gardner T. Improving estimates of neighborhood change with constant tract boundaries. Applied Geography. 2021 Jul;132:102476. 10.1016/j.apgeog.2021.102476 PMC8194102 34121782

[111] Consumer Financial Protection Bureau (CFPB). Manufactured-housing Consumer Finance in the United States. 2014.

[112] Crowder KD. The Racial Context of Residential Mobility: Neighborhood Conditions and Metropolitan Constraints. Department of Sociology, University at Albany, State University of New York; 1997. (PhD Dissertation).

[113] Mac Freddie. Single Family Loan-Level Dataset [Internet]. 2023. Available from: https://www.freddiemac.com/research/datasets/sf-loanlevel-dataset

[114] Mae Fannie. Loan Performance Data [Internet]. 2023. Available from: http://www.fanniemae.com/portal/funding-themarket/data/loan-performance-data.html

[115] Securities U.S. & Exchange Commission (SEC). EDGAR: Company Filings Lookup [Internet]. Available from: http://www.sec.gov/edgar/searchedgar/companysearch.html.

[116] Hyra DS, Squires GD, Renner RN, Kirk DS. Metropolitan Segregation and the Subprime Lending Crisis. Housing Policy Debate. 2013 Jan;23(1):177–98.

[117] Rugh JS. Why Latinos Were Hit Hardest by the U.S. Housing Crisis. Washington, DC: Urban Institute; 2012.

[118] Breen R, Karlson KB, Holm A. Interpreting and Understanding Logits, Probits, and Other Nonlinear Probability Models. Annu Rev Sociol. 2018 Jul 30;44(1):39–54.

[119] Garner CL, Raudenbush SW. Neighborhood Effects on Educational Attainment: A Multilevel Analysis. Sociology of Education. 1991 Oct;64(4):251.

[120] Sampson RJ, Raudenbush SW, Earls F. Neighborhoods and Violent Crime: A Multilevel Study of Collective Efficacy. Science. 1997 Aug 15;277(5328):918–24. doi: 10.1126/science.277.5328.918 9252316

[121] Raudenbush SW, Byrk AS. Hierarchical Linear Models. London: Sage Publications. Sage Publications; 2002.

[122] Hox J, Moerbeek M, Van de Schoot R. Multilevel analysis: Techniques and applications. Routledge; 2017.

[123] Guo G, Zhao H. Multilevel Modeling for Binary Data. Annu Rev Sociol. 2000 Aug;26(1):441–62.

[124] O’Neil Kuebler M. Lending in the Modern Era: Does Racial Composition of Neighborhoods Matter When Individuals Seek Home Financing? A Pilot Study in New England. City & Community. 2012 Mar;11(1):31–50.

[125] Desmond M. Evicted: Poverty and Profit in an American City. Maryland: Crown Publishers; 2016.

[126] Flippen CA. Residential Segregation and Minority Home Ownership. Social Science Research. 2001 Sep;30(3):337–62.

[127] Leong N. Racial Capitalism. SSRN Journal [Internet]. 2012 [cited 2024 Mar 29]; Available from: http://www.ssrn.com/abstract=2009877

[128] Melamed Jodi. Racial Capitalism. Critical Ethnic Studies. 2015;1(1):76.

[129] McMillan Cottom T. Where Platform Capitalism and Racial Capitalism Meet: The Sociology of Race and Racism in the Digital Society. Sociology of Race and Ethnicity. 2020 Oct;6(4):441–9.

[130] Robinson CJ. Black Marxism the Making of the Black Radical Tradition. Chapel Hill NC: University of North Carolina; 1983.

[131] Seamster L, Ray V. Against Teleology in the Study of Race: Toward the Abolition of the Progress Paradigm. Sociological Theory. 2018 Dec;36(4):315–42.

[132] Tilly C. Durable Inequality. Berkeley: University of California Press; 1999.

