# Supplementary material for: Racial/Ethnic inequality & contemporary disparities in mortgage lending
Source: PLoS One. 2025 Jan 14;20(1):e0308121. doi: 10.1371/journal.pone.0308121 (PMC11731762; doi:10.1371/journal.pone.0308121)
Supplement: S1 Table — (PDF) [file pone.0308121.s001.pdf]

S1 Table

	2004	2010
African American	-.914(.006)***	-.966 (0.14)***
Hispanic	-.428(.007)***	-.611(.014)***
Asian	.056(.011)***	-.044(.014)***
(Ref: White)		
Constant	1.310(.003)***	1.224(.004)***
Psuedo R^2	.019***	.013***
